# Combined Use of *N*-Palmitoyl-Glycine-Histidine Gel and Several Penetration Enhancers on the Skin Permeation and Concentration of Metronidazole

**DOI:** 10.3390/pharmaceutics10040163

**Published:** 2018-09-20

**Authors:** Sabrina Dahlizar, Mika Futaki, Akie Okada, Chihiro Yatomi, Hiroaki Todo, Kenji Sugibayashi

**Affiliations:** 1 Graduate School of Pharmaceutical Sciences, Josai University, Saitama 350-0295, Japan; sabrina043@yahoo.com (S.D.); gkm1818@josai.ac.jp (M.F.); gkm1706@josai.ac.jp (A.O.); ht-todo@josai.ac.jp (H.T.); 2 Department of Pharmacy, Faculty of Health Science, Syarif Hidayatullah State Islamic University Jakarta, Banten 15419, Indonesia; 3 Faculty of Pharmacy and Pharmaceutical Sciences, Josai University, Saitama 350-0295, Japan; c.metamon23@gmail.com

**Keywords:** Pal-GH, low molecular weight gelator, gel, skin permeation, enhancer, metronidazole

## Abstract

*N*-Palmitoyl-Glycine-Histidine (Pal-GH) is a novel low molecular weight gelator. In our previous report, ivermectin, a lipophilic drug, was effectively delivered to skin tissue after topical application with Pal-GH as a spray gel formulation, and a much higher skin concentration was confirmed than with the administration of a conventional oral formulation. The objective of this study was to increase the skin permeation of metronidazole (MTZ), a hydrophilic drug, after the topical application of Pal-GH gel. An evaluation of the combined effect of chemical penetration enhancers (CPEs), such as isopropyl myristate (IPM), propylene glycol (PG), ethanol, diethylene glycol monoethyl ether, and dimethyl sulfoxide (DMSO), on skin permeation was also conducted. We found that a 5% Pal-GH gel containing 1% MTZ (F5_MTZ_) exhibited a 2.7-fold higher MTZ permeation through excised hairless rat skin than its solution. Furthermore, F5_PG-MTZ_ and F5_IPM-MTZ_ further increased the skin permeation of MTZ when compared to F5_MTZ._ Interestingly, F5_PG-MTZ_ enhanced the skin penetration of MTZ, although no enhancement effect was observed for an MTZ solution containing PG. Thus, a Pal-GH formulation containing PG and IPM may enhance the skin permeation of MTZ.

## 1. Introduction

Low molecular weight gelators have been considered attractive novel soft materials over the past two decades. One such material, *N*-Palmitoyl-Glycine-Histidine (Pal-GH) was developed by Nissan Chemical Industries (Tokyo, Japan) and exhibits thixotropic behavior and high dissolving properties for a wide range of hydrophilic to lipophilic drugs. We reported on the usefulness of Pal-GH gel as a spray gel topical formulation base for the application of ivermectin [1]. An effective ivermectin concentration in the skin (more than 40 to 80 ng/g) was observed following the topical application. In addition, the Pal-GH spray gel showed high spreadability and low flowability on the applied skin site, suggesting that it may be a good formulation base for transdermal drug delivery.

Hairless rat skin has been used as a good alternative membrane for human skin to evaluate skin permeation profiles and the permeability coefficients of drugs [2,3,4,5]. Moreover, as the availability of sufficient human skin for skin permeation studies is usually unavailable due to ethical issues, hairless rat skin was used in this study. Although skin is broadly utilized as an application site for many drugs with expected topical and systemic pharmacological effects, the uppermost skin layer, the stratum corneum, has a high barrier property against skin permeation by drugs. Thus, it is a formidable challenge to enhance the skin permeation of poorly skin-penetrating drugs such as water-soluble drugs [6,7].

The use of chemical penetration enhancers (CPEs) has been extensively studied in the last three to four decades [8] to achieve suitable skin permeation and/or local concentration of topically applied drugs. Many types of CPEs, such as alcohols, fatty acid esters, ether alcohols, and sulfoxides, have been evaluated mainly by in vitro skin permeation experiments. However, the skin permeation of Pal-GH gel and the combination of several CPEs has not been studied. In the present study, metronidazole (MTZ, molecular weight; 171.15 g/mol, logK_o/w_; −0.15), a hydrophilic drug with low skin permeability, was selected as the model drug, and propylene glycol (PG) [9,10,11,12], ethanol (EtOH) [13], isopropyl myristate (IPM) [14], diethylene glycol monoethyl ether (Transcutol^®^) [15], and dimethyl sulfoxide (DMSO) [16] were selected as CPEs to represent alcohols, fatty acid esters, ether alcohols, and sulfoxides, respectively. DMSO can increase the lipid fluidity and promote drug partition [17,18,19,20,21]. PG [22,23,24] is known to solubilize α-keratin in the corneocytes, which is related to the intercellular penetration of drugs [25,26]. IPM directly acts on the stratum corneum, permeates into the lipid bilayer, and increases the fluidity of membranes [14,27]. Transcutol^®^ is a powerful solubilizing agent and an attractive penetration enhancer due to its nontoxicity. EtOH extracts lipids to increase the lipid fluidity, enhances drug solubility in stratum corneum lipids, causes changes in skin hydration and alterations in keratinized proteins, and has a drug solvent effect. The skin concentration of MTZ was also evaluated, as it is closely related to the pharmacological and/or toxicological effects of MTZ.

## 2. Materials and Methods 

### 2.1. Materials

Pal-GH premix (composed of 6% Pal-GH, 30% 1,2-octanediol, 20% 1,3-butanediol, 2% polyoxyethylene lauryl ether, 1% stearic acid, and 41% purified water) and propylene glycol acetate (PGA) were obtained from Nissan Chemical Industries, Ltd. (Tokyo, Japan). MTZ was purchased from Tokyo Chemical Industry (Tokyo, Japan). PG was purchased from Kanto Chemical Co. Inc. (Tokyo, Japan). EtOH, Transcutol^®^, DMSO, and IPM were purchased from Wako Pure Chemicals Industries, Ltd. (Osaka, Japan). Other chemicals and reagents were of special grade or HPLC grade, purchased from Wako Pure Chemical Industries, Ltd. (Osaka, Japan), and were used without further purification. 

### 2.2. Animals

Male hairless rats (WBM/ILA-Ht, 8 weeks of age, body weight of 200–250 g) were obtained from the Life Science Research Center, Josai University (Sakado, Saitama, Japan) or Ishikawa Experiment Animal Laboratories (Fukaya, Saitama, Japan). The animals were housed in temperature-controlled rooms (25 ± 2 °C) with a 12 h light-dark cycle (7:00–19:00 h). The rats were allowed free access to food (Oriental Yeast Co., Tokyo, Japan) and tap water. All animal feeding and breeding procedures, and the experiments themselves were approved by the Institutional Animal Care and Use Committee of Josai University (6 April 2017) with approval number H29003.

### 2.3. Preparation of Applied Formulations 

The Pal-GH formulations were prepared by a heating-and-cooling method as follows: Pal-GH premix was stirred with a stirrer bar on a magnetic stirrer (RSH-IDN, AS ONE Corporation, Osaka, Japan) in purified water and maintained at 85 °C until completely dissolved. Then, MTZ (100 mg) was dissolved in the Pal-GH solution at 75 °C, followed by the addition of several CPEs (IPM, PG, DMSO, EtOH, and Transcutol^®^). Furthermore, a PGA aqueous solution was prepared by stirring in purified water at 85 °C for 1 h. The obtained Pal-GH solution was mixed with the PGA solution at 1000 rpm and 75 °C for 1 h (until homogenous). The Pal-GH gel formulation was kept at room temperature prior to use. Pal-GH gel formulations without CPE were also prepared. In addition to the Pal-GH formulation, 1% MTZ solutions with and without CPE were also prepared to investigate the skin penetration enhancement effects from an aqueous solution. MTZ was completely dissolved in purified water and then CPE was added to the solution. The concentration of CPE in the MTZ solution was the same as that in the Pal-GH formulation. Table 1 shows the compositions of the Pal-GH formulations and MTZ solution with or without a chemical enhancer.

### 2.4. In Vitro Skin Permeation Experiment 

Full thickness hairless rat skin was excised from previously-shaved abdomens under intraperitoneally-administered anesthesia (medetomidine, 0.375 mg/kg; butorphanol, 2.5 mg/kg; and midazolam, 2 mg/kg). The excess fat was trimmed off and the skin was set in a Franz diffusion cell (effective diffusion area: 1.77 cm^2^) with the epidermis side facing the donor cell. The receiver cell was filled with 6.0 mL of phosphate buffered saline (PBS, pH 7.4), and 1 mL of PBS was applied to the epidermis side to reach an equilibration state for about 1 h. After an hour, the PBS of the epidermis side was replaced with the same volume of donor (1.0 mL of MTZ solution or Pal-GH formulation) to commence the permeation experiment. The temperature was maintained at 32 °C for 24 h. At predetermined times, an aliquot (0.5 mL) was withdrawn from the receiver chamber, and the same volume of fresh PBS was added to keep the volume constant. The obtained sample was used to measure the MTZ concentration by HPLC.

A side-by-side two-chamber diffusion cell with an effective permeation area of 0.95 cm^2^ was used for the application of the MTZ solution containing IPM due to the low solubility of IPM in the aqueous solution. The MTZ solution containing the IPM suspension (3.0 mL) was applied to the epidermal side, and the same volume of PBS was applied to the dermal side. Each solution was constantly stirred throughout the experiment to maintain the homogeneity of the donor solution (IPM in water) with a stirrer bar and kept at 32 °C by water circulation in a chamber jacket. At predetermined times, an aliquot (0.5 mL) was withdrawn from the dermal side, and the same volume of fresh PBS was added to keep the volume constant. In vitro skin permeation parameter (flux, permeability coefficient, and lag time) were calculated from the slope of the steady state portion of the amount of permeated drug. 

### 2.5. In Vitro Release Experiment

The MTZ release over 6 h was evaluated from the Pal-GH gel formulations (F2.5_MTZ_, F5_MTZ_, F10_MTZ_) and Pal-GH formulations containing CPEs. A dialysis membrane (molecular weight cut-off; 2000–14,000 Da, Sanko Junyaku Co., Tokyo, Japan) was soaked in PBS before being set in a vertical-type diffusion cell (effective diffusion area 0.95 cm^2^), and the receiver chamber was maintained at 32 °C. The receiver cell was filled with 6 mL of PBS, and 1.0 mL of Pal-GH formulation was applied to the donor cell while the receiver solution was agitated at 500 rpm using a magnetic stirrer. An aliquot (0.5 mL) was withdrawn from the receiver chamber, and the same volume of PBS was added to the chamber to keep the volume constant. The amount of MTZ released was determined. The release rate was calculated with the Higuchi equation.

### 2.6. Evaluation of Rheological Properties for N-Palmitoyl-Glycine-Histidine (Pal-GH) Formulations

The rheological properties of the Pal-GH formulations were evaluated using a rotational viscometer (RE-215H, Toki Sangyo Co., Ltd., Tokyo, Japan). The rotation speed was increased from 0 to 100 rpm over 7.5 min and decreased from 100 to 0 rpm over an equal time period, and the temperature was maintained at 32 °C. The corn rotor was 3° × R14, sample volume was 0.4 mL, and a flow analysis method was used. The hysteresis loop, thixotropic index, and yield value of the Pal-GH gel formulations were evaluated. The hysteresis area and yield value were calculated using VA 2000 software (Toki Sangyo Co., Ltd., Tokyo, Japan), whereas the thixotropic index was calculated by dividing the viscosity at 10 rpm by the viscosity at 100 rpm from the upward curve. 

### 2.7. Skin Concentration of Metronidazole (MTZ)

The skin concentrations of MTZ were evaluated after the application of selected Pal-GH formulations. At the end of the skin permeation experiment, the donor solution was removed using a cotton swab, and the skin sample was washed 10 times on the epidermis side and three times on the dermis side with 1 mL of PBS. The skin was blotted dry with Kimwipes^®^ paper. The compound-applied area was cut out and divided into parts for full thickness and viable epidermis and dermis (VED). For the measurement of the MTZ concentration in VED, the stratum corneum was removed by tape-stripping 20 times from the full-thickness skin using an adhesive tape (Cellotape^®^, Nichiban, Tokyo, Japan).

The MTZ concentrations in the full-thickness skin as well as VED were determined, and the amount of MTZ in the stratum corneum was calculated by subtracting the amount in the VED from that in the full-thickness skin. Then, the MTZ concentration in the stratum corneum was estimated under the assumption that the density and the thickness of the stratum corneum would be 1.2 g/cm^3^ [28] and 15.4 µm, respectively. The obtained skin piece (0.05 g) was reduced in size using scissors and homogenized at 12,000 rpm (4 °C, 5 min) with 0.45 mL of methanol for 2.5 min using a homogenizer (Polytron PT-MR 3000; Kinematica Inc., Littaue-Lucerne, Switzerland). Methanol (0.5 mL) was added and homogenized again for 2.5 min, followed by agitation at 32 °C for 15 min. Then, the mixture was centrifuged at 5000 rpm (4 °C, 5 min). The supernatant (100 µL) was mixed with the same volume of methanol and then agitated and centrifuged again under the same conditions. Finally, the obtained sample was used to detect the MTZ concentration by HPLC.

### 2.8. Determination of MTZ 

The obtained supernatant (20 µL) was injected into an HPLC system and measured under the following conditions.

The HPLC system (Shimadzu, Kyoto, Japan) consisted of a system controller (CBM-20A), pump (LC-20AD), degasser (DGU-20A_3_), auto-injector (SIL-20AC), a column oven (CTO-20A), UV detector (SPD-M20A), and analysis software (LC Solution). The column was a Superiorex^®^ ODS (5 µm, 4.6 × 250 mm) (Shiseido, Fine Chemicals; Tokyo, Japan), which was maintained at 40 °C. The mobile phase was methanol:water = 4:6 *v*/*v* (0–7 min). The flow rate was adjusted to 1.0 mL/min. The MTZ was detected at UV 254 nm. Briefly, the receiving solutions were mixed with the same volume of methanol and vortexed. After centrifugation at 21,500× *g* at 4 °C for 5 min, the resulting supernatant (20 μL) was injected directly into the HPLC system. 

### 2.9. Small Angle X-ray Scattering Analysis (SAXS)

X-ray diffraction analyses were performed with a small-angle X-ray diffraction system (Nano-Viewer, Rigaku Corporation, Tokyo, Japan), which was equipped with an X-ray generator (CuKα radiation, *λ* = 1.5418 Å), at a scanning rate of 5°. Diffraction analyses were performed using a vacuum-resistant glass capillary cell at 25 °C for 1 h. The scattering intensity was normalized by the decayed direct beam intensity. 

### 2.10. Transmission Electron Microscopy (TEM) 

A hydrogel sample was diluted 20 times with purified water and placed on a carbon-coated copper grid (400-Cu grids). Then, the samples were stained with 2% uranyl acetate. Transmission electron microscopic (TEM) images were recorded by JEM-1200EX (JEOL, Tokyo, Japan). The observations were carried out at an acceleration voltage of 80 kV. The observations were performed at the Hanaichi Ultrastructure Research Institute (Okazaki, Aichi, Japan).

### 2.11. Statistical Analysis

Statistical analyses of the skin permeation, in vitro release, and skin concentration of MTZ were performed using one-way ANOVA and Tukey’s Honestly Significant Different (HSD) post hoc analyses, and *p*-values less than 0.05 were considered to be significant.

## 3. Results

### 3.1. Physical Properties of Pal-GH Gel Formulation

**Figure 1:**
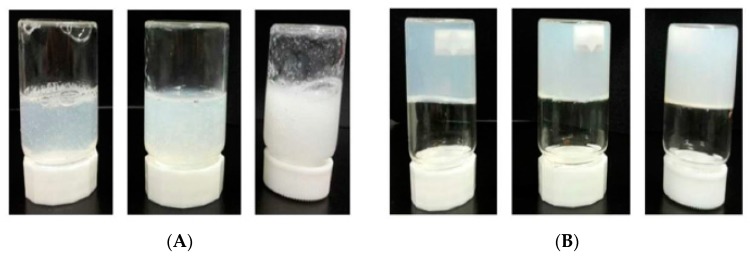
Photographs of the state of the *N*-Palmitoyl-Glycine-Histidine (Pal-GH) gel formulation in the bottles. The bottles were placed in an inverse position immediately (**A**) and 5 min after (**B**) shaking. The Pal-GH concentrations are, from left to right in both photos, 2.5%, 5%, and 10%.

Figure 1 shows the state of the Pal-GH gel formulation in the bottles. The bottles were put in an inverse direction immediately (Figure 1A) and after 5 min (Figure 1B) of being shaken at room temperature were tapped 10 times. F2.5_MTZ_, F5_MTZ_, and F10_MTZ_ are shown from left to right in both Figure 1A,B. These formulations showed a sol-gel transition within 5 min. 

### 3.2. In Vitro Skin Permeation of MTZ from Aqueous Solution

**Figure 2:**
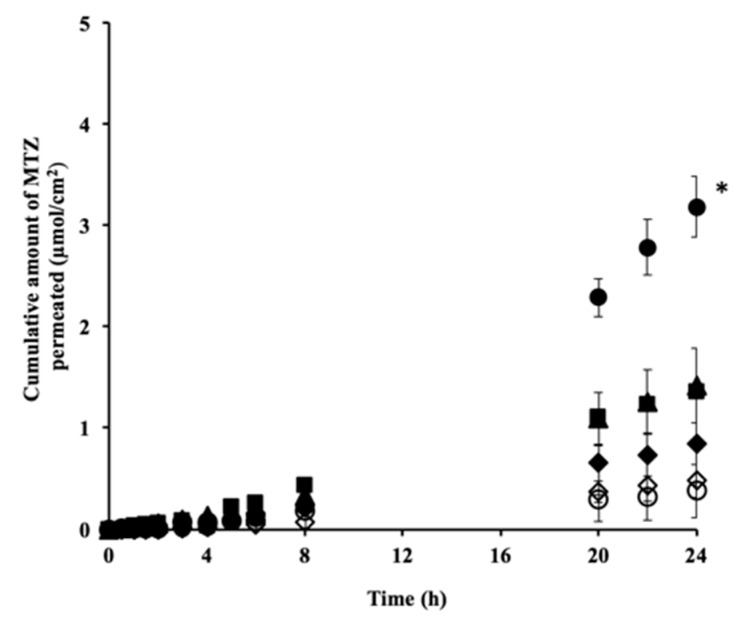
Permeation profiles of metronidazole (MTZ) through the excised hairless rat skin from its aqueous solution with or without a chemical enhancer. MTZ permeation was significantly (* *p* < 0.05) improved by isopropyl myristate (IPM) when compared with the aqueous solution without an enhancer (control) and other chemical penetration enhancers (CPEs). Symbols: ▲: aqueous solution, ●: IPM_MTZ_, ■: propylene glycol (PG_MTZ_), ◆: dimethyl sulfoxide (DMSO_MTZ_), ◇: ethanol (EtOH_MTZ_), ○: TRANS_MTZ_.

Figure 2 shows the permeation profiles of MTZ through excised hairless rat skin from the aqueous solution with or without CPE. The MTZ solution without CPE was used as a control. Only IPM_MTZ_ was associated with a skin penetration enhancement effect when compared with the control, and no skin penetration enhancement effects were observed in the other CPEs. PG_MTZ_ exhibited a similar MTZ permeation profile to the control, whereas TRANS_MTZ_ and EtOH_MTZ_ showed dramatically decreased permeation profiles when compared with the control.

### 3.3. In Vitro Release and Skin Permeation of MTZ from Pal-GH Gels 

**Figure 3:**
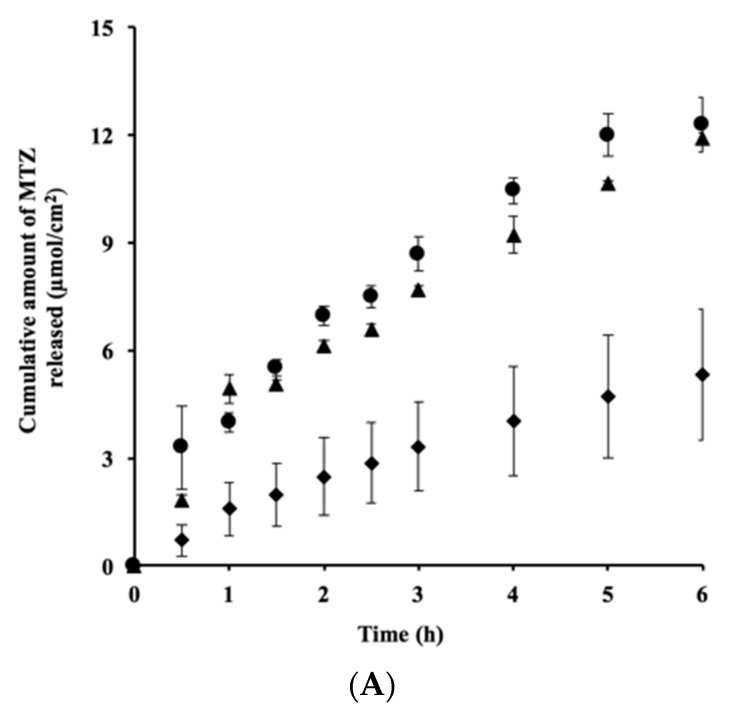

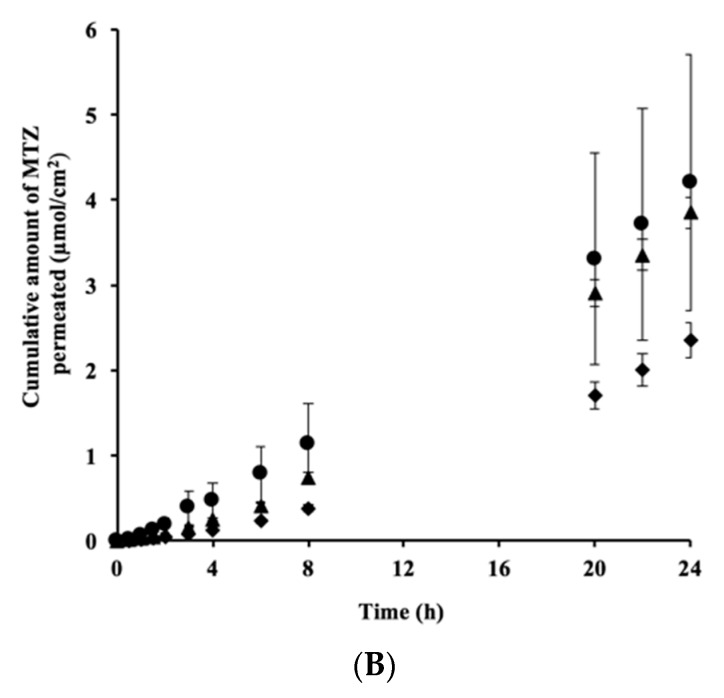
In vitro release (**A**) and skin permeation (**B**) of MTZ from different concentrations of Pal-GH gels. Symbols: ●: F2.5_MTZ_, ▲: F5_MTZ_, ◆: F10_MTZ_ (mean ± S.E., *n* = 4).

**Figure A1:**
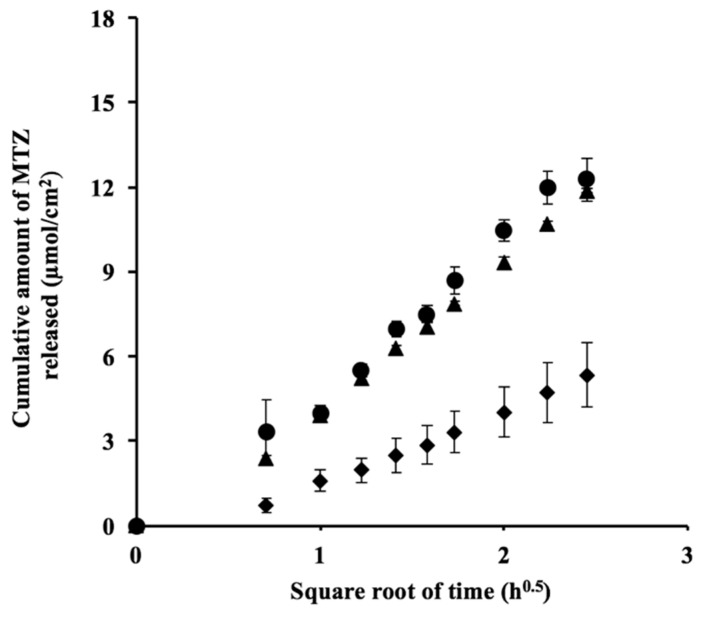
Higuchi plot analysis of the in vitro release of MTZ from different concentrations of Pal-GH gels. Symbols: ●: F2.5_MTZ_, ▲: F5_MTZ_, ◆: F10_MTZ_ (Mean ± S.E., *n* = 4).

Figure 3A shows the MTZ release profiles following exposure to different concentrations of Pal-GH gel formulation. The levels of MTZ release from F2.5_MTZ_ and F5_MTZ_ were similar, whereas that from F10_MTZ_ was lower. Furthermore, when the amounts of these releases were plotted against the squared root of time, straight lines were observed regardless of the Pal-GH concentration (Figure A1). 

Figure 3B shows the effect of the Pal-GH concentration on the time course of the cumulative amount of MTZ permeation through hairless rat skin. The cumulative levels of MTZ permeation over 24 h from F2.5_MTZ_ (Q_24h_; 4.2 µmol/cm^2^) and F5_MTZ_ (Q_24h_; 3.8 µmol/cm^2^) were higher than that from F10_MTZ_ (Q_24h_; 2.4 µmol/cm^2^). F2.5_MTZ_ showed a large coefficient of variation (*CV*) (>0.7) for all sampling points, whereas F5_MTZ_ and F10_MTZ_ had CVs < 0.3 for all sampling points. Thus, the F5_MTZ_ formulation was used to evaluate the effect of CPEs on the skin permeation of MTZ.

F5_MTZ_, F5_IPM-MTZ_, and F5_PG-MTZ_ showed higher release rates when compared with other formulations. Significant differences in release rates were observed between all formulations except between F5_MTZ_ and F5_IPM-MTZ_ (*p* < 0.05) (Table 2). 

**Figure 4:**
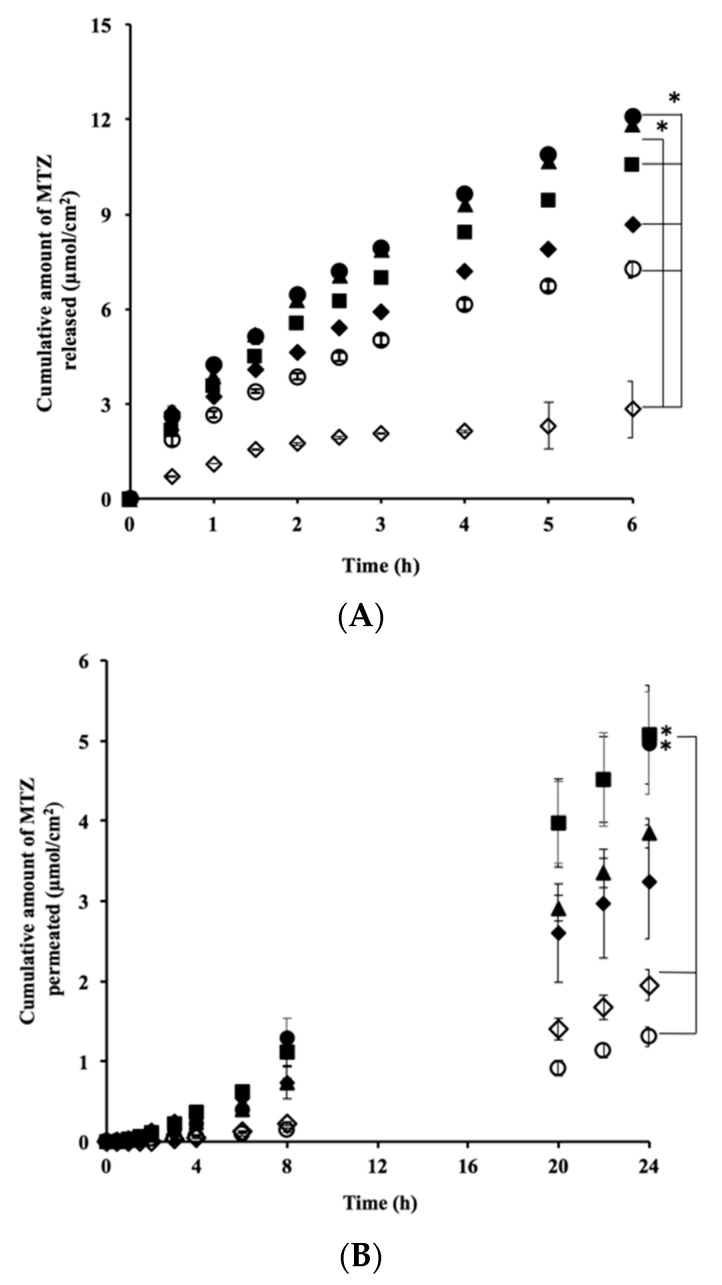
The effect of CPEs in Pal-GH gel formulations on the MTZ release (**A**) and skin permeation (**B**) profiles. Significant differences in MTZ release were observed among all formulations except between F5_MTZ_ and F5_IPM-MTZ_ (* *p* < 0.05), and in MTZ permeation between F5_PG-MTZ_ and F5_EtOH-MTZ_ or F5_TRANS-MTZ_; and between F5_IPM-MTZ_ and F5_EtOH-MTZ_ or F5_TRANS-MTZ_ (* *p* < 0.05). Symbols: ■: F5_PG-MTZ_, ●: F5_IPM-MTZ_, ▲: F5_MTZ_, ◆: F5_DMSO-MTZ_, ◇: F5_EtOH-MTZ_, ○: F5_TRANS-MTZ_ (Mean ± S.E., *n* = 3–4).

Figure 4A shows the effects of the CPEs in the Pal-GH gel formulations on the MTZ release profile. Compared with F5_MTZ_, F5_IPM-MTZ_ and F5_PG-MTZ_ had similar profiles, whereas F5_DMSO-MTZ_, F5_EtOH-MT_, and F5_TRANS-MTZ_ had significantly (*p* < 0.05) lower release profiles. Figure 4B shows the effect of the CPEs in the Pal-GH gel formulations on the time course of cumulative MTZ permeation. MTZ permeation from F5_IPM-MTZ_ and F5_PG-MTZ_ improved compared to that of F5_MTZ_. In contrast, the levels of MTZ permeation from F5_DMSO-MTZ_, F5_EtOH-MT_, and F5_TRANS-MTZ_ were lower than from F5_MTZ_. However, F5_DMSO-MTZ_ and F5_EtOH-MTZ_ showed higher levels, and F5_TRANS-MTZ_ exhibited a lower level of permeation compared to the aqueous solution (1% MTZ solution) (Figure 2). Pal-GH gels that showed higher levels of MTZ release (F2.5_MTZ_ ≅ F5_MTZ_ > F10_MTZ_) exhibited higher levels of skin permeation (F2.5_MTZ_ ≅ F5_MTZ_ > F10_MTZ_), as shown in Figure 3, but the release order from the Pal-GH gels containing CPEs did not match that obtained from the permeation results.

Table 3 summarizes the permeation fluxes, permeability coefficients, and lag times that were calculated from the skin permeation profiles. F5_IPM-MTZ_ and F5_PG-MTZ_ showed higher fluxes and permeability coefficients compared with the other formulations. Significant differences in the fluxes and permeability coefficients were also observed between F5_IPM-MTZ_ and F5_TRANS-MTZ_ or F5_EtOH-MTZ_ as well as between F5_PG-MTZ_ with F5_TRANS-MTZ_ or F5_EtOH-MTZ_.

### 3.4. Skin Disposition of MTZ after Topical Application of Pal-GH Formulations

**Figure 5:**
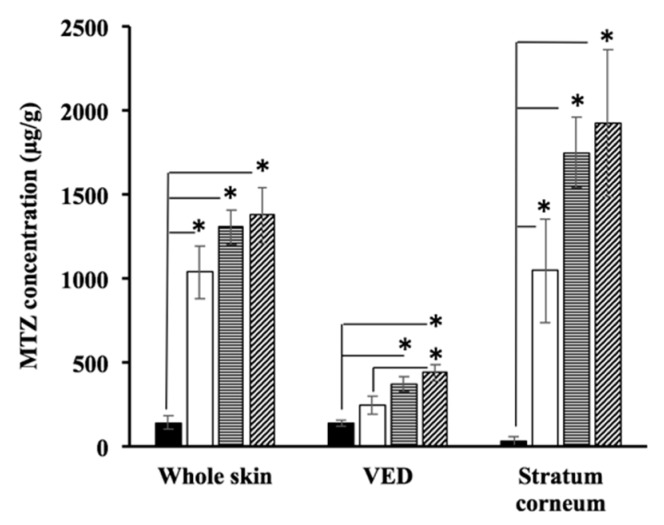
MTZ concentrations in the skin as a whole, the viable epidermis and dermis (VED), and the stratum corneum after the topical application of Pal-GH gel formulations on hairless rat skin. Significant differences were observed between F5_MTZ_, F5_PG-MTZ_, F5_IPM-MTZ_, and 1% solution of MTZ in the whole skin, and stratum corneum (* *p* < 0.05). Additionally, significant differences were observed between F5_PG-MTZ_ and F5_IPM-MTZ_ with a 1% solution of MTZ and between F5_IPM-MTZ_ with F5_MTZ_ in the VED (* *p* < 0.05). The MTZ concentration of the stratum corneum was estimated by dividing the MTZ difference (between the VED and whole skin) by the weight of the stratum corneum. Closed columns: 1% MTZ solution, open columns: F5_MTZ_, horizontal striped columns: F5_PG-MTZ_, striped columns: F5_IPM-MTZ_.

Figure 5 shows the skin concentration of MTZ after the in vitro skin permeation experiment had been completed (24 h). A topical application of 1% MTZ solution was used for comparison (control). MTZ concentrations were increased throughout the skin samples in the following order: control, F5_MTZ_, F5_PG-MTZ_, and then F5_IPM-MTZ_. This order was also observed in both the stratum corneum and the VED. In addition, the enhancement ratios of the MTZ concentration in the skin as a whole after the application of F5_MTZ_, F5_PG-MTZ_, and F5_IPM-MTZ_ were 7.2, 9.1, and 9.5-fold, respectively, versus that obtained from the control, and F5_PG-MTZ_ and F5_IPM-MTZ_ had 1.2 and 1.3-fold higher concentrations, respectively, compared to F5_MTZ_. In contrast, 30, 50, and 55-fold higher concentrations were obtained in the stratum corneum after the application of F5_MTZ_, F5_PG-MTZ_, and F5_IPM-MTZ_, respectively, compared to the control, and F5_PG-MTZ_ and F5_IPM-MTZ_ had 1.6 and 1.8-fold higher concentrations, respectively, compared to F5_MTZ_. Meanwhile, 1.8, 2.6, and 3.1-fold higher concentrations were achieved in the VED after the application of F5_MTZ_, F5_PG-MTZ_, and F5_IPM-MTZ_, respectively, and 1.5 and 1.7-fold higher concentrations were obtained for F5_PG-MTZ_ and F5_IPM-MTZ_, respectively, compared to F5_MTZ_.

### 3.5. Thixotropic Properties of Pal-GH Gel Formulations

**Figure 6:**
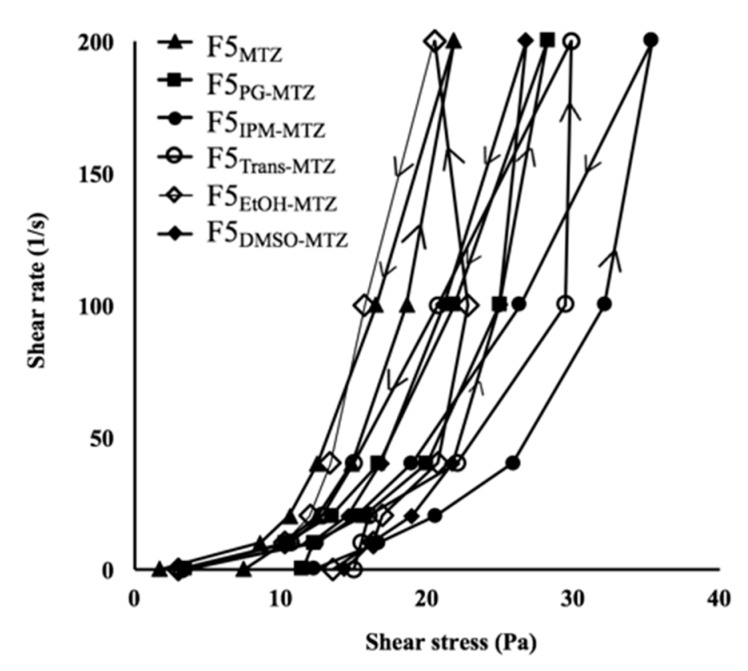
Thixotropic properties of the Pal-GH gel formulations.

The thixotropic properties of Pal-GH gel formulations were investigated. Table 4 and Figure 6 show the time-dependent viscosity changes of F5_MTZ_, F5_PG-MTZ_, F5_IPM-MTZ_, F5_TRANS-MTZ_, F5_EtOH-MTZ_, and F5_DMSO-MTZ_. The thixotropic behaviors were changed with the addition of CPE to the formulation. In particular, the area of the hysteresis loops of F5_PG-MTZ_ (945 Pa/s), F5_IPM-MTZ_ (1805 Pa/s), F5_TRANS-MTZ_ (2102 Pa/s), F5_EtOH-MTZ_ (2777 Pa/s), and F5_DMSO-MTZ_ (1241 Pa/s) increased compared to that of F5_MTZ_ (775 Pa/s). The obtained thixotropic index values of F5_PG-MTZ_ (5.57), F5_IPM-MTZ_ (5.83), and F5_TRANS-MTZ_ (5.35) were similar to that of F5_MTZ_ (5.71), whereas the thixotropic index values of F5_EtOH-MTZ_ (8.29) and F5_DMSO-MTZ_ (7.1) were higher than that of F5_MTZ_. In addition, the yield values of these formulations also changed, and the values for F5_PG-MTZ_, F5_IPM-MTZ_, F5_TRANS-MTZ_, F5_EtOH-MTZ_, and F5_DMSO-MTZ_ increased to 11, 13, 13, 14, and 14 Pa, respectively, from the F5_MTZ_ value of 8.0 Pa.

### 3.6. TEM Observations

**Figure 7:**
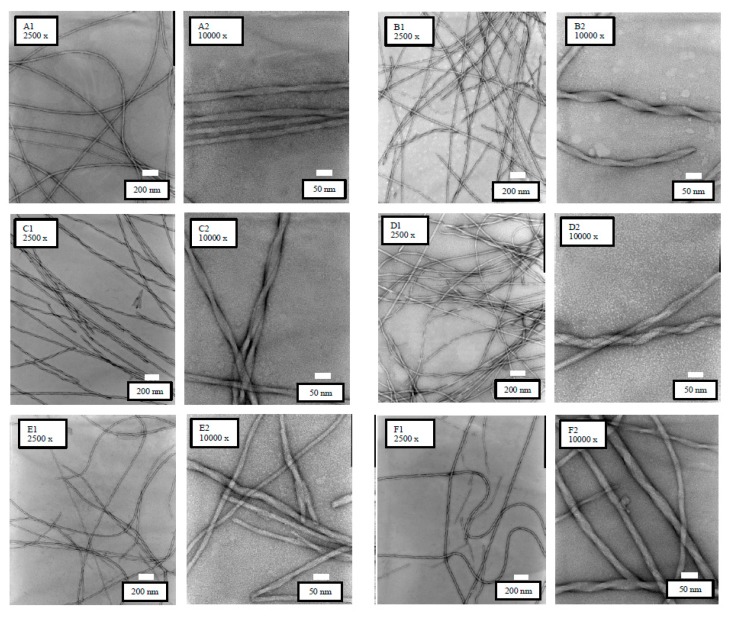
TEM observation results of the Pal-GH gel formulations at different magnifications (×2500 and ×10,000). F5_MTZ_ (A1,A2), F5_IPM-MTZ_ (B1,B2), F5_PG-MTZ_ (C1,C2), F5_DMSO-MTZ_ (D1,D2), F5_EtOH-MTZ_ (E1,E2), and F5_TRANS-MTZ_ (F1,F2).

Figure 7 shows the TEM images of the Pal-GH gel formulations. Similar images were observed from all formulations. However, F5_MTZ_, F5_EtOH-MTZ_, and F5_TRANS-MTZ_ showed homogeneous and straight fibrous structures, while F5_IPM-MTZ_, F5_PG-MTZ_, and F5_DMSO-MTZ_ had straight, but twisted, fibrous structures. 

## 4. Discussion

**Figure A2:**
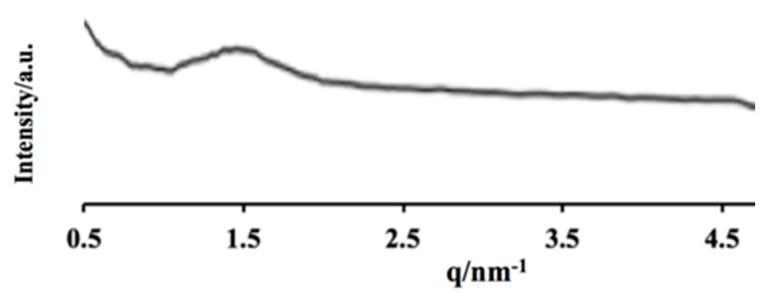
Small Angle X-ray Scattering Analysis (SAXS) profiles of F5_MTZ_. Curve is normalized and is horizontally shifted for clarity.

The skin permeation of MTZ was improved by the Pal-GH gel formulation. Supramolecular hydrogels with three-dimensional network structures can be formed through physical enlargement of nanofiber assemblies composed of amphiphilic molecules. Many published reports have shown that three-dimensional structures, such as micelles, lamella, and non-lamella, are useful drug carriers that can increase the skin permeation of entrapped drugs. A broad scattering SAXS pattern was confirmed for F5_MTZ_ (Figure A2 in Appendix A). Generally, micelle-like assemblies that are formed by amphiphilic molecules show a broadening peak [29]. Thus, the micelle-like assemblies in Pal-GH gels may act as drug carriers and enhance the skin permeation of MTZ.

Interestingly, the skin permeation of MTZ from F10_MTZ_ was lower than those from F2.5_MTZ_ and F5_MTZ_ (Figure 3B). The drug release profile from F10_MTZ_ was lower than those of F2.5_MTZ_ and F5_MTZ_ (Figure 3A). Since the skin permeation of MTZ must be related to its release profile, the lower MTZ release may be a reason for lower MTZ permeation from F10_MTZ_. MTZ release from F2.5_MTZ_, F5_MTZ_, and F10_MTZ_ can be explained by the Higuchi equation, suggesting that the drug was homogenously distributed in the gel. 

Compared to F5_MTZ_, similar MTZ release profiles were observed in F5_IPM-MTZ_ and F5_PG-MTZ_ (Figure 4A). On the other hand, F5_EtOH-MTZ_, F5_TRANS-MTZ_, and F5_DMSO-MTZ_ showed lower levels of MTZ release (Table 2). It would be reasonable to conclude that MTZ’s skin permeation profile and release are related, as shown in Figure 3. However, the MTZ release order from Pal-GH containing CPE did not correspond to its skin permeation order, suggesting that the effects of CPE on skin barrier function might be associated with higher skin permeation of MTZ. 

Among the CPEs, the IPM containing formulation, F5_IPM-MTZ,_ and the PG containing one, F5_PG-MTZ_, showed higher levels of MTZ skin permeation than F5_MTZ_ (Figure 4B). These results support our finding that the permeability coefficients of F5_IPM-MTZ_ and F5_PG-MTZ_ were the highest (Table 3). Similar lag times were observed for all formulations, indicating that CPEs do not change the barrier function of the SC. Therefore, F5_IPM-MTZ_ and F5_PG-MTZ_ could increase the skin permeation of MTZ by increasing the drug partition into the SC from gel. On the other hand, no skin permeation enhancement was observed in the other CPEs containing Pal-GH formulations (F5_EtOH-MTZ_, F5_TRANS-MTZ_, and F5_DMSO-MTZ_) when compared to F5_MTZ_. Since the penetration enhancement effect was only observed in IPM_MTZ_ in the present study (Figure 2), it may be reasonable to conclude that F5_PG-MTZ_ has a penetration enhancement effect. PG_MTZ_, DMSO_MTZ_, and EtOH_MTZ_ did not display this effect; however, the levels of MTZ permeation from F5_PG-MTZ_, F5_DMSO-MTZ_, and F5_EtOH-MTZ_ were 3.6, 2.3, and 1.3-fold higher than that of the control solution. The Pal-GH gel had a fibrous structure in all formulations. Thus, the construction of fibrous micelle-like Pal-GH assemblies may be a reason for the increase in MTZ permeation from the gel. Due to the forms of self-assembled structures, like micelles, they can fuse with the lipid bilayers of the stratum corneum, thereby enhancing the partitioning of the entrapped drug as well as the disruption of the ordered bilayer structure [30]. The levels of MTZ release from F5_DMSO-MTZ_, F5_EtOH-MTZ_, and F5_TRANS-MTZ_ were lower than that from F5_MTZ_. Since the skin permeation of a drug is related to its release from a formulation, a lower MTZ release should be related to lower skin permeation. These results are supported by the flux and release rate calculations shown in Table 2 and Table 3.

TEM observations were conducted to confirm the changes in the structures of the Pal-GH assemblies following the addition of several enhancers. The TEM observation results showed that the addition of PG, IPM, and DMSO to the Pal-GH formulation induced changes in the morphology from straight to twisted structures (Figure 7B–D). These results suggest that the structural differences of fibers may be related to the skin permeation enhancement effect of the entrapped drug in the Pal-GH gel formulation. Furthermore, the thixotropic behavior of the Pal-GH gel was changed by the addition of CPEs. This behavior could be related to the microstructure of Pal-GH assemblies in the formulation. The increasing formulation velocity is likely to be related to the network structures of micelle-like Pal-GH assembly because molecular–molecular interaction affects gel structure stabilization [31]. Highly dense and twisted fibrous structures were seen to be correlated with the thixotropic behavior of Pal-GH gel formulations. Further experiments are required to clarify the reasons for the changing rheological behavior and enhancement effect by CPEs incorporated in Pal-GH gel formulations. 

The treatment of many dermatological disorders relies on the penetration ability of active agents into the SC layer from applied formulations and their concentration in the viable epidermis and dermis layer [2]. The skin concentrations of MTZ were determined in the epidermis and dermis layer. The skin concentration of a topically applied drug has a very close relationship to its skin permeation [32], and in the steady-state, it can be expressed with the product of the partition coefficient and the drug concentration. Thus, the increased MTZ concentration in VED in F5_PG-MTZ_ and F5_IPM-MTZ_ was derived from increased drug partitioning from the formulation into the stratum corneum. The enhancement ratios of MTZ concentration in the whole skin and in the SC were much higher than those in VED. This may be related to the difficulty in completely removing the topically applied formulation from the applied site. Thus, the MTZ concentration in skin as a whole and in the SC may be overestimated by the remaining drug formulation on the skin surface. 

The present study shed light on the possibility of Pal-GH gel formulations enhancing the skin permeation and concentration. However, detailed reasons for the enhancement mechanism in skin permeation following the application of Pal-GH gel formulations compared to those containing CPEs were not clearly revealed. Further studies, currently underway, are needed to understand the skin permeation enhancement effect in hydrophilic drugs by Pal-GH gels.

## 5. Conclusions

In the present study, the skin permeation and concentration of MTZ improved following the application of Pal-GH as a formulation base. These results suggest that Pal-GH may have the potential to be used in dermatological formulations to improve their therapeutic efficacy, although elucidation of the enhancement mechanisms of Pal-GH gel and its constituent CPEs is necessary. 

## Figures and Tables

**Table 1:** Composition of the *N*-Palmitoyl-Glycine-Histidine (Pal-GH) gel formulations and metronidazole (MTZ) solution with or without a chemical enhancer prepared in this experiment. IPM = isopropyl myristate; PG = propylene glycol; ethanol; DMSO = dimethyl sulfoxide; and TRANS = isopropyl myristate.

Formulation Code	Pal-GH Conc. (Premix)	Chemical Penetration Enhancer
F2.5_MTZ_	2.5%	No enhancer
F5_MTZ_	5%	No enhancer
F5_IPM-MTZ_		IPM 10%
F5_PG-MTZ_		Propylene glycol 4%
F5_DMSO-MTZ_		DMSO 5%
F5_EtOH-MTZ_		Ethanol 20%
F5_TRANS-MTZ_		isopropyl myristate 10%
F10_MTZ_	10%	No enhancer
Aqueous solution		No enhancer
IPM_MTZ_		IPM 10%
PG_MTZ_		Propylene glycol 4%
DMSO_MTZ_		DMSO 5%
EtOH_MTZ_		Ethanol 20%
TRANS_MTZ_		Transcutol 10%

**Table 2:** Release rates of Pal-GH gel formulations.

Formulation Code	Release Rate (*K*) (µmol/cm^2^/h^0.5^)
F5_MTZ_	4.99 ± 0.99
F5_IPM-MTZ_	5.07 ± 0.09
F5_PG-MTZ_	4.43 ± 0.07
F5_DMSO-MTZ_	3.53 ± 0.05
F5_EtOH-MTZ_	1.11 ± 0.09
F5_TRANS-MTZ_	3.06 ± 0.11

Significant differences in the release rates were observed between all formulations except between F5_MTZ_ and F5_IPM-MTZ_ (*p* < 0.05).

**Table 3:** Fluxes, permeability coefficients, and lag times of the Pal-GH gel formulations.

Formulation Code	Flux (µg/cm^2^/h)	Permeability Coefficient (×10^−7^ cm/s)	Lag Time (h)
F5_MTZ_	17.7 ± 1.3	5.02 ± 0.37	1.59 ± 0.09
F5_IPM-MTZ_ *^,#^	29.44 ± 7.5	8.43 ± 2.14	1.61 ± 023
F5_PG-MTZ_ *^,#^	26.8 ± 4.79	7.69 ± 1.37	1.47 ± 0.21
F5_DMSO-MTZ_	17.08 ± 4.73	4.82 ± 1.33	1.60 ± 0.44
F5_EtOH-MTZ_	5.68 ± 1.01	1.59 ± 0.28	2.1 ± 0.23
F5_TRANS-MTZ_	3.54 ± 0.79	0.99 ± 0.22	1.86 ± 0.2

Significant difference (*p* < 0.05) between F5_IPM-MTZ_ and F5_TRANS-MTZ_ or F5_EtOH-MTZ_, F5_PG-MTZ_, and F5_TRANS-MTZ_ or F5_EtOH-MTZ_ for flux ^(^*^)^ and permeability coefficients ^(#)^.

**Table 4:** Viscosities of the Pal-GH gel formulations.

	Viscosity (mPa·s)											
rpm	F5_-MTZ_		F5_IPM-MTZ_		F5_PG-MTZ_		F5_DMSO-MTZ_		F5_EtOH-MTZ_		F5_TRANS-MTZ_	
	Upward	Downward	Upward	Downward	Upward	Downward	Upward	Downward	Upward	Downward	Upward	Downward
0.01	3.75 × 10^5^	8.75 × 10^4^	6.12 × 10^5^	1.68 × 10^5^	5.75 × 10^5^	1.75 × 10^5^	7.19 × 10^5^	1.81 × 10^5^	6.81 × 10^5^	1.50 × 10^5^	7.50 × 10^5^	1.62 × 10^5^
5	1.02 × 10^3^	8.62 × 10^2^	1.67 × 10^3^	1.25 × 10^3^	1.22 × 10^3^	1.04 × 10^3^	1.62 × 10^3^	1.21 × 10^3^	1.63 × 10^3^	1.02 × 10^3^	1.55 × 10^3^	1.07 × 10^3^
10	6.25 × 10^2^	5.31 × 10^2^	1.03 × 10^3^	7.62 × 10^2^	7.87 × 10^2^	6.75 × 10^2^	9.50 × 10^2^	7.31 × 10^2^	8.50 × 10^2^	6.00 × 10^2^	8.01 × 10^2^	6.44 × 10^2^
20	3.72 × 10^2^	3.12 × 10^2^	6.47 × 10^2^	4.75 × 10^2^	5.01 × 10^2^	4.18 × 10^2^	5.44 × 10^2^	4.22 × 10^2^	5.19 × 10^2^	3.34 × 10^2^	5.53 × 10^2^	3.75 × 10^2^
50	1.86 × 10^2^	1.65 × 10^2^	3.21 × 10^2^	2.63 × 10^2^	2.50 × 10^2^	2.18 × 10^2^	2.50 × 10^2^	2.11 × 10^2^	2.27 × 10^2^	1.57 × 10^2^	2.95 × 10^2^	2.07 × 10^2^
100	1.09 × 10^2^	1.09 × 10^2^	1.76 × 10^2^	1.76 × 10^2^	1.41 × 10^2^	1.41 × 10^2^	1.33 × 10^2^	1.33 × 10^2^	1.02 × 10^2^	1.02 × 10^2^	1.49 × 10^2^	1.49 × 10^2^

## References

[1] Sabrina D., Futaki M., Okada A., Kadhum W.R., Todo H., Sugibayashi K. (2018). Design of a topically applied gel spray formulation with ivermectin using a novel low molecular weight gelling agent, palmitoyl-glycine-histidine, to treat scabies. Chem. Pharm. Bull..

[2] Watanabe T., Hasegawa T., Takahashi H., Ishibashi T., Takayama K., Sugibayashi K. (2011). Utility of three-dimensional cultured human skin model as a tool to evaluate skin permeation of drugs. Altern. Anim. Test Exp..

[3] Morimoto Y., Hatanaka T., Sugibayashi K., Omiya H. (1992). Prediction of skin permeability of drugs: comparison of human and hairless rat skin. J. Pharm. Pharmacol..

[4] Abd E., Yousef S.A., Pastore M.N., Telaprolu K., Mohammed Y.H., Namjoshi S., Grice J.E., Roberts M.S. (2016). Skin models for testing of transdermal drugs. Clin. Pharmacol. Adv. Appl..

[5] Jung E.C., Maibach H.I. (2015). Animal models for percutaneous absorption. J. Appl. Toxicol..

[6] Cross S.E., Roberts M.S. (2000). The effect of occlusion on the epidermal penetration of parabens from a commercial test ointment, acetone and ethanol vehicles. J. Investig. Dermatol..

[7] Asbill C.S., Michniak B.B. (2000). Percutaneous penetration enhancers: Local versus transdermal activity. Pharm. Sci. Technol..

[8] Asbill C.S., El-Kattan A.F., Michniak B. (2000). Enhancement of transdermal drug delivery: Chemical and physical approaches. Crit. Rev. Ther. Drug Carrier Syst..

[9] Barrett C.W., Hadgraft J.W., Caron G.A., Sarkany I. (1965). The effect of particle size on the percutaneous absorption of fluocinolon acetonide. Br. J. Dermatol..

[10] Coldman M.F., Poulsen B.J., Higuchi T. (1969). Enhancement of percutaneous absorption by the use of volatile: nonvolatile system as vehicles. J. Pharm. Sci..

[11] Hoelgaard A., Mollgaard B. (1985). Dermal drug delivery-improvement by choice of vehicle or drug derivative. J. Control. Release.

[12] Nicolazzo J.A., Morgan T.M., Reed B.L., Finnin B.C. (2005). Synergistic enhancement of testosterone transdermal delivery. J. Control. Release.

[13] Kurihara-Bergstrom T., Knutson K., DeNoble L., Goates C.Y. (1990). Percutaneous absorption enhancement of an ionic molecule by ethanol-water systems in human skin. Pharm. Res..

[14] Leopold C.S., Lippold B.C. (1995). An attempt to clarify the mechanism of the penetration enhancing effects of lipophilic vehicles with differential scanning calorimetry (DSC). J. Pharm. Pharmacol..

[15] Harrison J.E., Watkinson A.C., Green D.M., Hadgraft J., Brain K. (1996). The relative effect of azone and transcutol on permeant diffusivity and solubility in human stratum corneum. Pharm. Res..

[16] Roth S.H., Fuller P. (2011). Diclofenac sodium topical solution 1.5% w/w with dimethyl sulphoxide compared with placebo for the treatment of osteoarthritis; pooledsafety results. Postgrad. Med..

[17] Thong H.Y., Zhai H., Maibach H.I. (2007). Percutaneous penetration enhancers: An overview. Skin Pharmacol. Physiol..

[18] Zafar S., Ali A., Aqil M., Ahad A. (2010). Transdermal drug delivery of labetalol hydrochloride: Feasibility and effect of penetration enhancers. J. Pharm. Bioallied. Sci..

[19] Marren K. (2011). Dimethyl sulfoxide: An effective penetration enhancer for topical administration of NSAIDs. Phys. Sportsmed..

[20] Scheuplein R.J., Blank I.H. (1971). Permeability of the skin. Physiol. Rev..

[21] Sekura D.L., Scala J. (1972). The percutaneous absorption of alkyl methyl sulfoxides. Adv. Biol. Skin..

[22] Mills P.C. (2007). Vehicle effects on the in vitro penetration of testosterone through equine skin. Vet. Res. Commun..

[23] Gee C.M., Watkinson A.C., Nicolazzo J.A., Finnin B.C. (2014). The effect of formulation excipients on the penetration and lateral diffusion of ibuprofen on and within the stratum corneum following topical application to humans. J. Pharm. Sci..

[24] Kietzmann M., Blume B. (1997). Percutaneus absorption of betamethasone from different formulations using the isolated perfused bovine udder. In Vitro Toxicol..

[25] Herai H., Gratieri T., Thomazine J.A., Bentley M.V., Lopez R.F. (2007). Doxorubicin skin penetration from monoolein-containing propylene glycol formulations. Int. J. Pharm..

[26] Mollgaard B., Hoelgaard A. (1983). Vehicle effect on topical drug delivery. I. Influence of glycols and drug concentration on skin transport. Acta Pharm. Suec..

[27] Hirvonen J., Rajala R., Vihervaara P., Laine E., Paronen P., Urtti A. (1994). Mechanism and reversibility of penetration enhancer action in the skin. Eur. J. Pharm. Biopharm..

[28] Anderson R.L., Cassidy J.M. (1973). Variation in physical dimension and chemical composition of human stratum corneum. J. Investig. Dermatol..

[29] Matsumoto K., Shundo A., Ohno M., Fujita S., Saruhashi K., Miyachi N., Miyaji K., Tanaka K. (2015). Modulation of physical properties of supramolecular hydrogels based on a hydrophobic core. Phys. Chem. Chem. Phys..

[30] Karande P., Mitragotri S. (2009). Enhancement of transdermal drug delivery via synergistic action of chemicals. Biochim. Biophys. Acta.

[31] Mallia V.A., Weiss R.G. (2016). Correlations between thixotropic and structural properties of molecular gels with crystalline networks. Soft Matter..

[32] Sugibayashi K., Todo H., Oshizaka T., Owada Y. (2010). Mathematical model to predict skin concentration of drugs: Toward utilization of silicone membrane to predict skin concentration of drugs as an animal testing alternative. Pharm. Res..

